# Detailed information gain and therapeutic impact of whole body computed tomography supplementary to conventional radiological diagnostics in blunt trauma emergency treatment: a consecutive trauma centre evaluation

**DOI:** 10.1007/s00068-020-01502-1

**Published:** 2020-09-30

**Authors:** Christian Tibor Josef Magyar, Franziska Maeder, Michael Diepers, Felix Amsler, Thomas Gross

**Affiliations:** 1 Trauma Unit, Kantonsspital Aarau, Tellstrasse 25, 5001 Aarau, Switzerland; 2 Division of Neuroradiology, Department of Radiology, Kantonsspital Aarau, Tellstrasse 25, 5001 Aarau, Switzerland; 3 Amsler Consulting, Gundeldingerrain 111, 4059 Basel, Switzerland

**Keywords:** Trauma, Acute Care Surgery, Whole body CT, Imaging, ER diagnostics, Computed tomography

## Abstract

**Purpose:**

The indication of whole body computed tomography (WBCT) in the emergency treatment of trauma is still under debate. We were interested in the detailed information gain obtained from WBCT following standardized conventional imaging (CI).

**Methods:**

Prospective study including all emergency trauma centre patients examined by CI (focused assessment of sonography in trauma, chest and pelvic X-ray) followed by WBCT from 2011 to 2017. Radiology reports were compared per patient for defined body regions for number and severity of injuries (Abbreviated Injury Scale, AIS; Injury Severity Score, ISS), incidental findings and treatment consequences (Wilcoxon signed rank test, Spearman rho, Chi-square).

**Results:**

1271 trauma patients (ISS 11.3) were included in this study. WBCT detected more injury findings than CI in the equivalent body regions (1.8 vs. 0.6; *p* < 0.001). In 44.4% of cases at least one finding was missed by CI alone. Compared to WBCT, injury severity of specified body regions was underestimated by CI on average by an AIS of 1.9 (p < 0.001). In 22.0% of cases injury severity increased by an AIS ≥ 2 following WBCT. In 16.8% of patients additional injury findings resulted in a change of treatment (number needed to profit, NNP = 6 patients): NNP decreased from 25 for patients with an ISS < 7 up to nearly 2 for patients with an ISS > 25 at final evaluation, thereby demonstrating a significant improvement in the NNP with increasing ISS (rho = 0.33, *p* < 0.001). Moreover, WBCT in 88.4% of patients identified ≥ 1 incidental finding (mean 3.4) vs. 28.9% by CI only (*p* < 0.001). Overall, WBCT had treatment consequences in 31.9% of cases (NNP = 3.1).

**Conclusions:**

The application of WBCT in addition to CI in the emergency treatment of trauma had therapy consequences for almost every third patient. On the other hand, WBCT appeared not to be indicated (ISS < 8) in at least 2/5 of patients.

**Electronic supplementary material:**

The online version of this article (10.1007/s00068-020-01502-1) contains supplementary material, which is available to authorized users.

## Introduction

Suspected major trauma requires urgent diagnostics, often followed by immediate lifesaving interventions. To commence appropriate emergency treatment, a straightforward and complete assessment of the injured patients is needed [1, 2]. One of the most commonly used approaches is the Advanced Trauma Life Support (ATLS) [1], including conventional imaging (CI) methods such as the Focused Assessment with Sonography for Trauma (FAST) and plain radiographs of the chest (CXR) and the pelvis (PXR). Computed tomography (CT) can be used as a diagnostic adjunct to the secondary survey of patients [1]. In many trauma centres so-called ‘whole body CT’ (WBCT; alternatively named ‘polytrauma CT’, ‘total body CT’ or ‘pan scan’) has become a standard specification, partially complementing, partially replacing the aforesaid basic radiology tools [3, 4].

Despite the increasing use of WBCT in the emergency room (ER) treatment of trauma, evidence is limited on the usefulness of WBCT, particularly in less severely injured patients. Depending on individual perspectives, the arguments pro and contra range from time saving to time loss [5, 6], risk of radiation [7, 8], obstructing direct care of the patient under treatment to possible life saving effects due to its merits as a quick and sensitive diagnostic tool [9]. Most of all, recommendations for its use differ importantly in the literature due to conflicting findings on the impact of WBCT on trauma patient survival [2, 6, 10–14]. Consequently, no consensus exists on the indication criteria for its use, when to execute WBCT only, when to add WBCT to conventional imaging, or any combinations.

Having practised the ATLS approach now for years in our hospital, we continued to execute basic radiological imaging first after the primary survey of patients, followed by the decision for or against additional WBCT, i.e., depending on trauma team activation criteria fulfilled and trauma leaders’ assessment of single cases. Screening the literature, we became aware of a lack of evidence, partially explained by missing detail in clinical data on the usefulness or overuse of WBCT compared to conventional radiological diagnostics [9, 15, 16]. A search for larger studies reporting the detailed comparison of CI with WBCT yielded no results relevant to our specific interest in the ‘knowledge benefit’ of WBCT per patient supplementary to well defined CI in terms of information on pathologies and the treatment consequences for patients. Surprisingly, precise sensitivity and specificity rates for the reported ER diagnostic procedures are missing. We, therefore, conducted this prospective, observational study in blunt trauma patients who had undergone ‘complete standard CI’ (FAST, CXR and PXR) followed by WBCT. Given the existing literature, the trial was intentionally not designed to investigate the potential impact of WBCT on other aspects, such as patient survival [2, 6, 10–14] or radiation exposure, but instead focused on three study aims:

First, to objectify the detailed differences in the identification of the number and severity of injuries found by WBCT per patient compared to defined single and ‘complete standard CI’ as performed routinely in the emergency treatment of trauma patients; second, to capture the number of non-trauma-related incidental findings from WBCT compared to CI; third, to identify both, the resulting therapeutic impact of WBCT on patient treatment compared to defined standard CI as well as the rate of unnecessary WBCT.

With this detailed investigation, we, therefore, intended to provide precise data, e.g., the currently missing sensitivity and specificity rates that result from the diagnostic procedure described above as to add more evidence relevant to the emergency treatment of the injured.

## Methods

### Study design and selection of patients

**Fig. 1:**
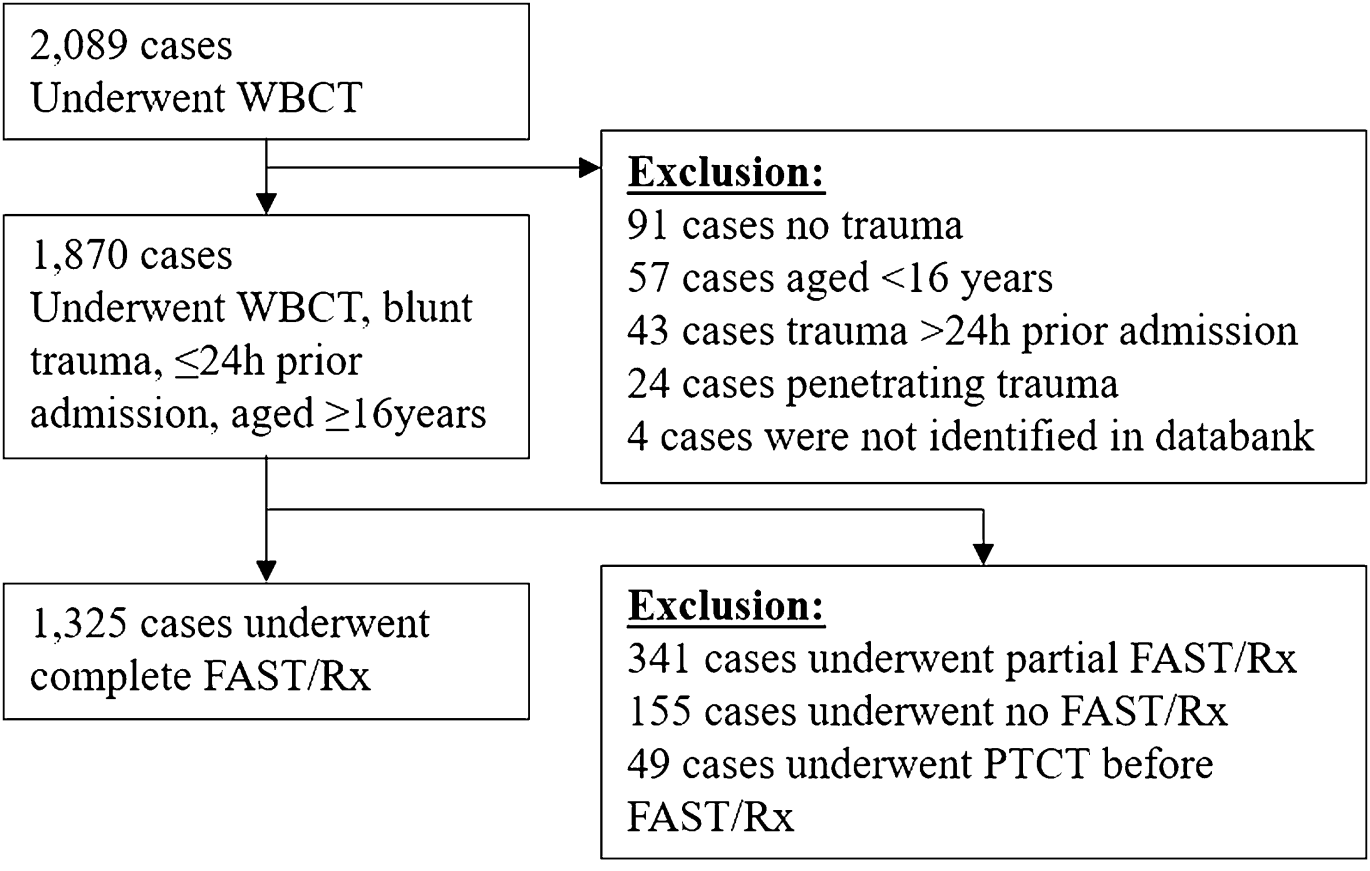
Study cohort flowchart

This prospective observational investigation from 1 st November 2011 to 31 st December 2017 took place in one of the 12 Swiss trauma centres officially designated for the treatment of highly specialized medicine (HSM). The study was approved by the local ethics committee (PB_2018-00079-AG/SO 2012-008; NCT registered). Trauma treatment followed ATLS^©^ standards [1] and ER team activation criteria (ERTAC) as published recently [17, 18], with a routine use of CI and a subsequent liberal application of WBCT as soon as ERTAC were fulfilled as the standard approach. The concrete execution depended on trauma leaders’ valuation of single cases. Study nurses not involved in the treatment of patients evaluated all consecutive WBCT imaging procedures undertaken at the hospital in patients aged ≥ 16 years within 24 h of traumatic blunt injury. All WBCT-patients who did not undergo the defined CI were excluded from the analysis (Fig. 1).

### Definitions

So-called ‘complete standard CI’ for this investigation was defined as the execution of all of the following three ER radiological examinations: focused assessment with sonography for trauma (FAST) performed by the resident radiologist on duty, and plain emergency radiographs (XR) of the chest (CXR) and pelvis (PXR), each with the patient supine and undertaken by radiology technicians. After CI, WBCT imaging followed for all patients using a standardized scanning protocol with the patient positioned supine, starting from the head and ending at the hip region, just distal to the trochanter minor, using the Toshiba Aquilion CXL (Toshiba Corp.^©^, Tokyo, Japan). Procedure began with a head and neck scan with both arms and hands positioned next to the body; in a second step the arms were positioned above the head, if possible, followed by two helical scans: first scan from frontal sinus to the first thoracic vertebral body, second helical scan from the shoulders to the distal trochanter minor region under biphasic contrast agent injection. A resident radiologist primarily evaluated the scans with an initial report. Within 24 h of the examination the images were reviewed by a senior radiologist and a definitive WBCT report was generated in all cases. For this investigation all definitive radiology reports were used for further evaluation and comparison of the results.

Findings in CI were compared to the findings of subsequent WBCT limited to the relevant body regions only: CXR to CT scan of the thorax (with and without the thoracic spine), FAST to CT scan of the abdomen (without the lumbar spine region) and PXR to CT scan of the pelvis. In addition, the results of combined CI were compared to the relevant combined CT-regions. The body regions head, neck and extremities (other than the hip and shoulder) were excluded from this evaluation, because they could not be evaluated by specified CI. Given the restricted value of plain emergency radiographs of the chest and pelvis for the assessment of spine, findings were elaborated for both, including and not including spine injuries in the comparison to WBCT reports. A radiological finding in WBCT was defined as ‘new’ or ‘missed’ if it had not already been identified by CI. The definition of ‘missed injuries’ was based on previous trauma reviews [19]. A finding was defined as false positive if it had been identified by CI before, but not in subsequent WBCT. Findings were categorized as injury-related or incidental (not trauma related). The abbreviated injury severity score (AIS) [20] and resultant Injury Severity Score (ISS) were applied in a standardized manner to determine the injury severity of single lesions or specified body regions, both for single radiological examinations and for the maximum (‘final’) information on patients’ injury sequelae at hospital discharge according to all records. A ‘partial ISS’ was calculated using AIS solely for the thorax, abdomen and pelvis. AIS and ISS-values were calculated by an Association for the Advancement of Automotive Medicine (AAAM) AIS-qualified study nurse using the relevant definitions of the AIS 2005 version of the TraumaRegister DGU^®^ of the German Trauma Society (https://www.traumaregister-dgu.de/index.php?id=433&L=1). The differences between CI and WBCT as measured on the AIS and ISS were categorized into 3 groups: (a) as ‘false high’ if FAST or XR findings were found to be more than one AIS point higher than verified in WBCT, (b) ‘false low’ if WBCT was more than one point higher than CI, and (c) ‘equal’ if AIS values did not differ more than one AIS point between imaging techniques. Further comparison of imaging techniques also included the number of patients requiring surgical procedures and/or emergency interventions, both overall and in single body regions. ‘Interventions’ were subclassified as (a) ‘immediate interventions’ (< 6 h following admittance) or (b) surgical procedures in the operation room > 6 h following hospital admittance (yes/no). ‘Immediate interventions’ were again subdivided into (a) ER interventions such as thoracic drainage or embolization and (b) surgical interventions in the operation room (OR) including craniotomy, thoracotomy, laparotomy, revascularization, stabilization of the pelvis and stabilization of extremities. Furthermore, interventions were grouped according to the body region involved based on the AIS study regions (thorax, abdomen and pelvis). Change of trauma treatment procedure was defined as any intervention resulting directly from new trauma-related findings diagnosed by WBCT but not by CI. A change in subsequent management based on incidental findings was recorded in cases, where additional diagnostic or therapeutic investigations were recommended by the radiologist in charge. Treatment consequences were defined as the sum of both a change of trauma treatment procedures and/or a change of further management based on incidental findings.

The degree of superiority in the number (*n*) and severity (AIS; ISS) of trauma-related findings diagnosed by WBCT compared to CI as well as treatment changes resulting from WBCT imaging were defined as measures of primary outcome. The rate of ‘unnecessary’ WBCT procedures, defined as WBCT for patients with a final ISS < 8 and/or the number of incidental findings diagnosed were defined as secondary outcome measures in this investigation.

### Statistical analysis

Results are presented as numbers and percentages or means and where appropriate medians with corresponding parameters (minimum, 25th percentile, median, 75% percentile and maximum). Missing data were excluded variable-wise. All statistical tests were two-tailed. Student’s *t* test for paired comparison was used to test for differences of means and chi-square analysis was used to test categorical data. Spearman correlations (rho) were calculated to test for the correspondence of conventional and WBCT diagnostics and to test for the influence of the ISS. Data were analysed using SPSS™ for Windows 24 (Armonk^©^, NY: IBM Corp, USA), and a *p-* value < 0.05 was considered significant.

## Results

2089 consecutive patients underwent WBCT in the study period. After exclusion of patients following defined criteria, 1271 emergency trauma cases remained (Fig. 1). Details of demographic, trauma and treatment characteristics are displayed in Table 1.

**Table 1:** Demographic and clinical characteristics of the study cohort (*n* = 1271)

Demographics	Mean (minimum, 25th percentile, median, 75th percentile, maximum)/*N* (%)
Age	49.2 (16;31;49;65;96)
Gender (female)	414 (32.6%)
**Injury mechanism** (*n* = 1266)	
High energy	897 (70.9%)
Traffic accident	684 (54.0%)
Fall height ≥ 3 m	213 (16.8%)
**Abbreviated injury scores (AIS)**	
AIS head & neck	1.38 (0;0;1;2;6)
AIS face	0.25 (0;0;0;0;4)
AIS chest	1.00 (0;0;0;2;5)
AIS chest without spine	0.90 (0;0;0;2;5)
AIS abdomen	0.47 (0;0;0;0;5)
AIS abdomen without spine	0.22 (0;0;0;0;5)
AIS extremities and pelvis	0.96 (0;0;0;2;5)
AIS upper extremities	0.47 (0;0;0;0;4)
AIS under extremities	0.43 (0;0;0;0;4)
AIS pelvis	0.92 (0;0;0;0;5)
AIS external lesions	0.59 (0;0;1;1;4)
Maximal AIS > 3	204 (16.1%)
**Injury severity score (ISS)**	
Total ISS at hospital discharge	11.3 (0;3;9;17;75)
ISS = 0	8 (0.6%)
ISS = 1–7	513 (40.4%)
ISS = 8–15	386 (30.4%)
ISS = 16–25	252 (19.8%)
ISS ≥ 26	112 (8.8%)
**Number of injury findings**	
Total number of injury findings detected in WBCT	3.1 (0;1;2;5;21)
**Clinical descriptives**	
Hospital mortality	83 (6.5%)
Length of stay (days)	9.0 (0;2;5;13;92)
Length of ICU stay (days; *n* = 496; 39.0%)	1.7 (0.1;0.9;1.9;5.0;81.5)
Any intervention (yes)	515 (40.5%)
Any intervention thorax, abdomen, pelvis only (with spine)	199 (15.7%)
Any intervention thorax, abdomen, pelvis only (without spine)	147 (11.6%)
Change of trauma treatment procedure (due to new WBCT findings)	213 (16.7%)
Overall treatment consequences (change of trauma treatment procedure or incidental finding with need for further management)	409 (31.9%)
**Immediate interventions thorax**	
Intercostal drainage	50 (3.9%)
Thoracotomy	1 (0.1%)
**Immediate interventions abdomen**	
Laparotomy	8 (0.6%)
**Immediate interventions pelvis**	
Pelvic stabilization	10 (0.8%)
**Other immediate interventions**	
Craniotomy	54 (4.2%)
Extremities stabilization	21 (1.7%)
Embolization	8 (0.6%)
Revascularization	3 (0.2%)
**Missed and incidental findings**	
Patients with at least one injury finding AIS > 0 in conventional diagnosis	394 (31.0%)
Patients with at least one injury finding AIS > 0 (abdomen, thorax, pelvis) in WBCT	584 (45.9%)
Patients for whom at least one injury finding was missed in conventional diagnosis compared to WBCT (abdomen, thorax, pelvis)	565 (44.5%)
Patients with at least one incidental finding in WBCT	1144 (90.0%)
Patients with at least one incidental finding in WBCT requiring further diagnostic/therapeutic management	222 (17.5%)

### Number and classification of trauma findings

In 69.7% of cases at least one injury finding was documented by WBCT. The number of injury related findings detected by WBCT ranged from 0 to 21 per case. Comparing WBCT vs. CI as defined according to equivalent body regions revealed that significantly more injury findings and more severe injuries (partial ISS) were detected by WBCT (Table 2).

**Table 2:** Comparison of the number of injury findings and the severity of injury between conventional imaging and WBCT in corresponding AIS body regions

	Conv. imaging^a^	WBCT^a^	Mean difference	*p* value	Spearman rho
**Overall**					
Number of findings (thorax, abdomen, pelvis, with spine)	0.6 (0;0;0;1;8)	1.8 (0;0;1;3;21)	1.2	< 0.001	0.66
Number of findings (thorax, abdomen, pelvis, without spine)	0.6 (0;0;0;1;8)	1.1 (0;0;0;2;14)	0.5	< 0.001	0.73
Partial ISS (thorax, abdomen, pelvis, with spine)	2.7 (0;0;0;4;34)	5.7 (0;0;4;9;57)	3.0	< 0.001	0.71
Partial ISS (thorax, abdomen, pelvis, without spine)	2.7 (0;0;0;4;34)	4.6 (0;0;0;9;57)	1.9	< 0.001	0.75
**Thorax (chest X-ray)**					
Number of findings chest	0.3 (0;0;0;0;6)	0.8 (0;0;0;1;9)	0.5	< 0.001	0.70
AIS chest	0.6 (0;0;0;0;5)	1.0 (0;0;0;2;5)	0.4	< 0.001	0.68
**Abdomen (FAST)**					
Number of findings abdomen	0.1 (0;0;0;0;2)	0.2 (0;0;0;0;4)	0.1	< 0.001	0.32
AIS abdomen	0.1 (0;0;0;0;4)	0.3 (0;0;0;0;5)	0.2	< 0.001	0.35
**Pelvis (pelvic X-ray)**					
Number of findings pelvis	0.2 (0;0;0;0;3)	0.2 (0;0;0;0;3)	0.0	0.242	0.85
AIS pelvis	0.3 (0;0;0;0;4)	0.4 (0;0;0;0;5)	0.1	< 0.001	0.87

^a^Mean (minimum; 25th percentile; median; 75% percentile; maximum)

Overall, in 24.1% (*n* = 306) injury findings were diagnosed both in CI and WBCT. In an additional 8.8% (*n* = 112) a suspected traumatic lesion was identified by CI, of which *n* = 19 could not be verified in WBCT. In contrast, WBCT in 73.7% (*n* = 937) found at least one injury-related finding (range 1–18). In an additional 2.0% (*n* = 26) findings were unclear, e.g., unexplained free fluid. Therefore, in 44.5% (*n* = 565) injury findings were missed by CI (Table 1).

With regard to patients’ final injury severity and restricted to findings per se as detected by WBCT, patients demonstrated a mean ISS of 9.3 (Table 1), whereas 41.0% (*n* = 521) had an ISS < 8 at discharge. Overall, 28.4% of cases (*n* = 361) yielded an AIS ≥ 2 in at least one of the body regions examined.

Comparing WBCT with CI of the thorax, abdomen and pelvis revealed a significantly higher mean injury severity detected by WBCT in all body regions (Table 2), with an overall mean difference of ISS 1.9. Table 3a–c show detailed differences between radiological modalities per body region with regard to diagnosed injury severity and subsequent surgical interventions. The thoracic region was found to have the highest discrepancy with regard to injury severity (AIS) as diagnosed by WBCT vs. CXR (0.4; Table 2). If the thoracic spine is included (vs. excluded) in the analysis of the thoracic region, then a false low AIS for CXR was found in 15.0% (vs. 4.2%) and a false high AIS in 1.3% (vs. 6.3%). 4.7% (*n* = 9) of the false low group had to undergo subsequent thoracic surgery (including the spine) vs. 6.3% (*n* = 1) of the false high group compared to overall 3.1% needing surgery (Table 3a). Analogous data for the abdominal and pelvic regions are given in Table 3b, c.

**Table 3:** a–c: Grouped differences in injury severity (AIS), grouped by false high, similar and false low results for classified body regions as diagnosed by conventional imaging and whole body computed tomography (WBCT), and subsequent surgical interventions

(a) Thoracic body region	Total		Categorized AIS on WBCT			Emerg. Inter-costal drainage (ICD)		Other thoracic surgery (excl. thoracic spine)		Surgery thoracic spine	
	*n*	%	0	1–2	3–5	*n*	%	*n*	%	*n*	%
AIS CXR > WBCT by > 1 (false high)	16	1.3	15	1	0	2	12.5	1	6.3	1	6.3
AIS CXR and WBCT ± 1	1064	83.7	791	87	186	39	3.7	30	2.8	31	2.9
AIS CXR < WBCT by < 1 (false low)	191	15.0	0	87	104	7	3.7	9	4.7	8	4.2
Total	1271	100.0	806	175	290	48	3.8	39	3.1	40	3.1
			63.4%	13.8%	22.8%						

(b) Abdominal body region	Total		Categorized AIS on WBCT			Laparotomy		Other abdominal intervention (excl. lumbar spine)	
	*n*	%	0	1–2	3–5	*n*	%	*n*	%
AIS FAST > WBCT by > 1 (false high)	23	1.8	21	2	0	1	4.3	1	4.3
AIS FAST and WBCT ± 1	1164	91.6	1087	58	19	5	0.4	16	1.4
AIS FAST < WBCT by < 1 (false low)	84	6.6	0	55	29	2	2.4	16	19.0
Total	1271	100.0	1108	115	48	8	0.6	33	2.6
			87.2%	9.0%	3.8%				

(c) Pelvic body region	Total		Categorized AIS on WBCT			Stabilisation of pelvis			Other pelvic intervention	
	*n*	%	0	1–2	3–5		*n*	%	*n*	%
AIS PXR > WBCT by > 1 (false high)	12	0.9	12	0	0		0	0.0	0	0.0
AIS PXR and WBCT within ± 1	1229	96.7	1089	43	97		10	0.8	35	2.8
AIS PXR < WBCT by < 1 (false low)	30	2.4	0	19	11		0	0.0	5	16.7
Total	1271	100.0	1101	62	108		10	0.8	40	3.1
			86.6%	4.9%	8.5%					

Additionally, the maximum AIS in any of the body regions thorax, abdomen and pelvis was calculated for the comparison of findings in defined CI vs. WBCT, in line with the gold standard of the final injury severity coding at hospital discharge (Table 4a). Concerning ‘partial ISS’ a significantly higher injury severity was found in WBCT vs. CI (mean difference 1.9; *p* < 0.001; Table 2).

**Table 4:** Comparison of the maximum difference in injury severity (AIS) in the three body regions abdomen, thorax and pelvis between WBCT and conventional imaging as diagnosed in conventional imaging vs. WBCT and subsequent surgical interventions according to final categorized injury severity of patients (ISS)

ISS		Total		Immediate interventions excluding head		Surgery in AIS-regions thorax, abdomen or pelvis only		Surgery in AIS-regions thorax, abdomen or pelvis only, excluding spine	
		*n*	%	*n*	%	*n*	%	*n*	%
0–7	AIS difference conv. imag. and WBCT ± 1	479	91.9	2	0.4	11	2.3	2	0.4
	AIS difference conv. imag. and WBCT > 1	42	8.1	0	0.0	1	2.4	1	2.4
	Total	521	100.0	2	0.4	12	2.3	3	0.6
8–15	AIS difference conv. imag. and WBCT ± 1	287	74.4	16	5.6	41	14.3	24	8.4
	AIS difference conv. imag. and WBCT > 1	99	25.6	2	2.0	16	16.2	12	12.1
	Total	386	100.0	18	4.7	57	14.8	36	9.3
16–25	AIS difference conv. imag. and WBCT ± 1	166	65.9	16	9.6	37	22.3	27	16.3
	AIS difference conv. imag. and WBCT > 1	86	34.1	8	9.3	32	37.2	27	31.4
	Total	252	100.0	24	9.5	69	27.4	54	21.4
> 25	AIS difference conv. imag. and WBCT ± 1	60	53.6	21	35.0	29	48.3	25	41.7
	AIS difference conv. imag. and WBCT > 1	52	46.4	24	46.2	32	61.5	29	55.8
	Total	112	100.0	45	40.2	61	54.5	54	48.2
Total	AIS difference conv. imag. and WBCT ± 1	992	78.0	55	5.5	118	11.9	78	7.9
	AIS difference conv. imag. and WBCT > 1	279	22.0	34	12.2	81	29.0	69	24.7
	Total	1271	100.0	89	7.0	199	15.7	147	11.6

Comparing the highest AIS of single body regions, i.e., the maximum difference in injury severity per body region between WBCT and CXR (Table 4b), 22.0% (*n* = 279) of cases demonstrated a false low result in CI in at least one of the three regions.

### Incidental findings

In 90.0% (*n* = 1144) of cases at least one incidental finding was diagnosed with WBCT (Table 1). On average 3.4 (range 0–15) incidental findings were detected per patient. WBCT identified significantly more incidental findings compared to CI (88.4% vs. 28.9%; *p* < 0.001). In 47.8% (*n* = 608) at least one incidental finding was only found in WBCT and not in CI. In 17.5% (*n* = 222) incidental findings were judged diagnostically as requiring further management (diagnostic or therapeutic), i.e., resulted in a recommendation by the radiology specialist for further diagnostic work-up or a follow-up inspection or was directly followed by a clinical intervention. In *n* = 87 of these cases (39%) it was recommended that further diagnostics should be performed by the patient’s general practitioner after discharge.

### AIS difference and interventions

40.5% of individuals underwent trauma-related interventions (Table 1), of which 199 cases (15.7%) involved the three investigated body regions (Table 3a–c) including the spine and 147 cases (11.6%) without the spine. Table 4 shows the maximum difference in injury severity of the three body regions. Overall, 22.0% (*n* = 279) showed a difference of at least 2 AIS-points in at least one of the three investigated body regions. The incidence for this diagnostic difference following WBCT increased significantly with increasing ISS (Spearman rho = 0.32, *p* < 0.001).

### Change of treatment and interventions

In 16.8% (*n* = 213) of all patients, trauma treatment was changed based on new injury findings detected by WBCT in the three body regions (Table 5) which corresponds to a ‘number needed to profit’ (NNP) of 6 patients. Within this group, 23.0% (*n* = 49) of treatments were performed in an emergency setting, 59.6% (*n* = 127) were operations performed later in the AIS regions thorax, abdomen or pelvis (including spinal involvement) vs. 38.5% (*n* = 82) without the spine. Studying more in detail the potential interrelation of early (pre-) clinical injury information (i.e., early trauma team activation criteria) with the subsequent information gain and/ or treatment change obtained by the execution of WBCT found only low correlations in univariate analysis (*r* < 0.25; Suppl. Table A). Table 5 shows additional data on resulting change of treatment and surgical emergency interventions with regard to final injury severity of patients (categorized ISS). The incidence for a change of treatment resulting from the execution of WBCT following CI increased significantly with increasing ISS (Spearman rho = 0.33, *p* < 0.001). Accordingly, the NNP from WBCT decreased from 25 for patients with an ISS < 7 up to nearly 2 for patients with an ISS > 25 at final evaluation.

**Table 5:** Comparison of change of trauma treatment based on new findings detected by WBCT in any of the three investigated body regions as diagnosed in conventional imaging vs. WBCT and by resulting surgical interventions with regard to final categorized injury severity of patients (ISS)

ISS		Total		Immediate interventions excluding head		Surgery in AIS-regions thorax, abdomen or pelvis only		Surgery in AIS-regions thorax, abdomen or pelvis only, excluding spine	
		*n*	%	*n*	%	*n*	%	*n*	%
0–7	None	499	95.8	1	0.2	3	0.6	2	0.4
	Change of trauma treatment	22	4.2	1	4.5	9	40.9	1	4.5
	Total	521	100.0	2	0.4	12	2.3	3	0.6
8–15	None	312	80.8	13	4.2	23	7.4	20	6.4
	Change of trauma treatment	74	19.2	5	6.8	34	45.9	16	21.6
	Total	386	100.0	18	4.7	57	14.8	36	9.3
16–25	None	185	73.4	10	5.4	25	13.5	22	11.9
	Change of trauma treatment	67	26.6	14	20.9	44	65.7	32	47.8
	Total	252	100.0	24	9.5	69	27.4	54	21.4
> 25	None	62	55.4	16	25.8	21	33.9	21	33.9
	Change of trauma treatment	50	44.6	29	58.0	40	80.0	33	66.0
	Total	112	100.0	45	40.2	61	54.5	54	48.2
Total	None	1058	83.2	40	3.8	72	6.8	65	6.1
	Change of trauma treatment	213	16.8	49	23.0	127	59.6	82	38.5
	Total	1271	100.0	89	7.0	199	15.7	147	11.6

In total, if incidental findings are included, there was at least one treatment consequence in 31.9% (*n* = 405) of cases as a result of new findings from WBCT, which corresponds to a NNP of 3.1 patients.

## Discussion

To the best of our knowledge, this study is the largest standardized prospective monocenter investigation to compare the results of WBCT with previous combined CI modalities in the management of emergency trauma cases. The evaluation yielded three major findings:

First, the routine use of WBCT resulted in significant information gain in comparison to single as well as ‘complete CI’, valid for both, injury and incidental findings: In almost every second patient at least one finding was missed by CI only. Our data correlate well with earlier publications reporting a poor correlation of clinical and conventional radiological diagnoses compared to injury-related findings detected by WBCT [5, 21–25]. A rate of 65% of undetected injuries in CXR compared to CT was reported in a smaller prospective study on blunt chest trauma [26]. With regard to the pelvis, another investigation in 2/3 of patients identified fractures in WBCT that had been missed on PXR [27]. Our investigation confirmed that defined CI significantly underestimated injury severity in the body regions under examination compared to WBCT. At first glance, the minor differences, for example, in the number of rib fractures diagnosed per patient or the average difference of about two ISS points per patient, allowing for higher false low and false high injury severity rates for CI, may not seem particularly impressive from a clinical point of view. But it has to be emphasized that so far such detailed data have not been published: Without WBCT a lesion in at least one of the examined body regions thorax, abdomen or pelvis would have been missed or its severity underestimated by at least two AIS-points in more than every fifth patient. In an important number of patients potentially life-threatening injury sequelae would have been overlooked without WBCT imaging. Similar findings were reported by Topp et al. with data from the German TraumaRegister DGU^®^ [28]. However, reported rates vary in the literature partly due to different policies on how to process tentative findings. On the other hand, the liberal use of WBCT in our study also resulted in an important overtriage: Patients’ final ISS was < 8 in 50% of cases if only the ISS coding of the body regions thorax, abdomen and pelvis were considered. In the literature reported overtriage rates vary importantly, for example, depending on the trauma cohort under investigation, i.e., single criteria for ER treatment or trauma team activation [17] and subsequent execution of WBCT [3] or the definition of which patients require WBCT [24]. A small retrospective study found a 32% ‘justified WBCT rate’, defined as multi-region injuries (> = 2 body regions with an AIS > 1) [4]. A German TraumaRegister DGU^®^ analysis verified an average overtriage rate of 32% using an (ex post) patient injury severity of ISS ≥ 15 as the indication that WBCT would be required [28]. Given the importance of AIS ≥ 3 lesions (and not only patients’ injury severity in total), we decided on an overtriage cut-off of ISS > 8 as benchmark in this study.

Second, with regard to incidental findings, WBCT in almost nine out of ten patients identified incidental findings, whereas CI did so in only three out of ten. Many of these findings might be irrelevant to the patient although WBCT findings did result in a need for further management in almost every fifth patient. In the literature the rate for incidental findings resulting from WBCT imaging for trauma patients ranges from 43 to 55% [9, 29–33]. One major reason for the higher rate of incidental findings found in our investigation compared to other authors might be the higher mean age of our study cohort [29–33].

Third, and most important from a clinical point of view, based on WBCT findings changes were made to the management of trauma and planned interventions in every sixth patient. With increasing ISS at final evaluation, the according NNP from WBCT following CI improved significantly, with a NNP of 25 for patients with an ISS < 7 to almost two for patients with an ISS > 25. Therefore, even less severely injured patients may relevantly benefit from the (additional) execution of WBCT in such an emergency scenario. Such numbers have to be viewed in the context of the fact that, for example, for patients taking statins as the most widely accepted standard prevention after stroke, the reported NNP amounts to only > 30 [34]. If resultant incidental findings were also taken into account, treatment consequences resulted in every third patient. We found no other study investigating and demonstrating similar differences between the use of CI vs. WBCT per patient. To date, published data only show partial comparisons, such as the usefulness of routine WBCT in blunt head trauma, the comparison of solely CXR vs. WBCT or selective CT of the thorax or abdomen compared to CXR and FAST, et alia [25, 26, 35, 36]. One might also argue that FAST anyway should only diagnose free abdominal liquid or pericardial tamponade. Modified CI, e.g., the use of extended FAST (including a thoracic evaluation in addition to routine abdomen and pericardium as is done in conventional FAST) might improve the observed differences somewhat, but not essentially, given our data compared to the therapeutic consequences in up to 10% of cases resulting from extended FAST vs. FAST as described by Zieleskiewicz et al. in their recent retrospective evaluation [37]. In our hospital, eFAST was only introduced in the routine management of trauma after the end of this investigation. The diagnostic and therapeutic usefulness of WBCT in the management of trauma in reality would have to be estimated as even higher if the body regions head and neck were also included in the study [25]. The rate of findings might be even greater if WBCT was also used to image the extremities rather than just the torso, but current techniques are associated with longer exams and higher radiation per case.

Currently, some hospitals already base their management of the critically ill on WBCT only [3], executing CI only in exceptional cases. Furthermore, new technologies promise to allow the effective use of WBCT from head to toe in less time and with lower radiation doses without decreasing diagnostic quality. Nevertheless, more cautious authors still find that WBCT should be reserved for those patients for whom clinicians have a high suspicion of extensive polytrauma. These authors emphasize the need for specific decision-making models and/or scores on which to base an imaging requirement [4]. In our eyes, the demonstrated relatively low NNP rates for WBCT even in less severely injured and the shown insufficient correlations of early (pre-) clinical criteria as to reliably indicate the need for subsequent WBCT argue against such an restricted use of WBCT in the management of the injured. Nevertheless, given the caveats as highlighted and the associated costs, continued critical appraisal of WBCT is needed, above all, with regard to the indication criteria. To date, independent from the advantages of WBCT described, the combined use of conventional and CT techniques, at least for the less severely injured, appears to still hold an important place. Furthermore, optimized local and organizational implementation of WBCT in the emergency diagnostics and treatment of the severely injured, such as in multifunctional or hybrid ERs or ORs, as published in recent years, for example, in Switzerland [38] or Japan [3], might offer additional benefits in the near future.

### Limitations

This study has several shortcomings. Even though data were acquired prospectively for all consecutive trauma WBCT cases, this investigation, following its study objective and subsequent comparative design, purposely only reports patients suffering blunt trauma who underwent both CI as defined and subsequent WBCT. If we had also included WBCT cases with incomplete CI imaging, the investigation would have been disadvantaged by a major number of missing cases in the detailed comparison of body regions. We excluded penetrating trauma from this study to homogenize the study population, because it is typically associated with different injuries and treatment regimens, and our cases are few. The decision to use WBCT in individual cases was taken by the trauma leader on duty, which could be seen as a potential confounder, even though internal hospital guidelines recommend its standard use as soon as trauma team activation criteria [17] were actually fulfilled after primary evaluation of the patient in the ER. The evaluation is restricted to the cohort investigated due to its monocenter design and, ultimately, includes more older or less severely injured than several other study groups [29–33]. Given established literature data [35] and own experience, WBCT was executed instead of sequential CTs of single body regions as soon as more than one region were suspected to be injured or following high energy trauma. Such procedure based on the observation, that with such an approach serial CTs may be avoided, less injury sequelae information will be lost, and radiation exposure could be avoided for patients indeed in need for CT. Given the main objective of the study, i.e., a somehow post-hoc view on the resulting impact of WBCT in the treatment of trauma patients compared to defined standard CI, this work did not preferentially investigate predictive clinical factors indicating WBCT in this context. The weak correlations found between tested trauma team activation criteria and investigated outcome variables further underline the complexity of such an alternative approach. Therefore, the study also did not analyse other clinical predictors (e.g., blood pressure) possibly meliorating the pre-test probability for the use of WBCT. Some readers may argue that the detailed differences identified may only be of practical value if there are clinical consequences. However, from an evidence-based approach and given the missing information to date, our study objective was to provide precise data, such as sensitivity and specificity rates for the described diagnostic procedure in the ER treatment of the injured.

## Conclusion

WBCT subsequent to standardized conventional imaging was found to add information in three out of four of patients in this series of blunt ER trauma cases. In almost half of cases more than one finding was missed by CI alone compared to WBCT, the latter leading to a change of treatment in almost 1/3 of patients. However, clinical indispensability was only demonstrated in about 1/5 of cases, highlighting the risk of overuse of this radiation tool in daily practice. Even though less severely injured patients less often profited from additional WBCT, still every 25^th^ patient with an final ISS < 8 showed a change of treatment resulting from the execution of WBCT. Given the increasing utilization of WBCT in the emergency management of trauma evidence-based guidelines are urgently needed.

## Electronic supplementary material

Below is the link to the electronic supplementary material.

Supplementary file1 (DOCX 40 kb)
