# Supplementary material for: Detailed information gain and therapeutic impact of whole body computed tomography supplementary to conventional radiological diagnostics in blunt trauma emergency treatment: a consecutive trauma centre evaluation
Source: Eur J Trauma Emerg Surg. 2020 Sep 30;48(2):921–31. doi: 10.1007/s00068-020-01502-1 (PMC9001527; doi:10.1007/s00068-020-01502-1)
Supplement: Supplementary file 1 — Supplementary file1 (DOCX 40 kb) [file 68_2020_1502_MOESM1_ESM.docx]

**Suppl. Table A:** Correlation of trauma team activation criteria^1^ with injury findings and the severity of injury found between conventional imaging and WBCT in corresponding AIS body regions (univariate analysis)

| Pearson | Total ISS at hospital discharge | Teil-ISS def CT (Thorax, Abdomen, Pelvis, with spine | Teil-ISS DGU (Thorax, Abdomen, Pelvis, without spine) | AIS head & neck | AIS Abdomen | AIS Abdomen without spine | AIS Chest | AIS Chest without spine | AIS Pelvis | Difference AIS Abdomen vs. FAST | Difference AIS Chest def CT vs. Rx | Difference AIS Pelvis im def CT vs. Rx | Difference any AIS (Abd, Chest, Pelvis) def CT vs. FAST/Rx | change of treatment due to new injury findings def CT vs FAST/Rx |
| --- | --- | --- | --- | --- | --- | --- | --- | --- | --- | --- | --- | --- | --- | --- |
| ***Demographic data*** |  |  |  |  |  |  |  |  |  |  |  |  |  |  |
| Age at injury | 0.22*** | 0.08** | 0.09*** | 0.23*** | -0.04 | -0.08** | 0.13*** | 0.15*** | 0.05 | -0.02 | 0.01 | 0.01 | 0 | 0.05 |
| Gender, female | -0.05 | -0.05 | -0.05 | 0.04 | 0.01 | -0.02 | -0.08** | -0.08** | 0.02 | 0.05 | -0.03 | -0.02 | 0.01 | -0.09*** |
| ***Transfer criterion*** |  |  |  |  |  |  |  |  |  |  |  |  |  |  |
| Admission from another hospital (secondary care) | 0.09*** | 0.04 | 0.03 | 0.16*** | 0.06* | 0.03 | 0.01 | -0.01 | 0.04 | 0.02 | 0 | 0.03 | 0.02 | 0 |
| ***Mechanism of injury*** |  |  |  |  |  |  |  |  |  |  |  |  |  |  |
| Traffic injury except car | 0.11*** | 0.07** | 0.07* | 0.04 | 0.03 | 0.04 | 0.09*** | 0.06* | 0.03 | 0.01 | -0.01 | -0.06* | -0.02 | 0.01 |
| High fall (≥ 3 m) | 0.07** | 0.08** | 0.07* | -0.04 | 0 | 0 | 0.05 | 0.05 | 0.1*** | -0.01 | 0.04 | 0.03 | 0.02 | 0.09*** |
| Mechanism of injury criteria (penetrating, traffic except car, fall ≥ 3 m) | 0.15*** | 0.13*** | 0.11*** | 0.01 | 0.03 | 0.03 | 0.12*** | 0.09*** | 0.1*** | 0 | 0.02 | -0.03 | 0 | 0.08** |
| ***Anatomic criterion*** |  |  |  |  |  |  |  |  |  |  |  |  |  |  |
| EMS injury | 0.38*** | 0.28*** | 0.3*** | 0.15*** | 0.15*** | 0.17*** | 0.27*** | 0.27*** | 0.15*** | 0.13*** | 0.09*** | 0.06* | 0.15*** | 0.19*** |
| ***Physiological criteria*** |  |  |  |  |  |  |  |  |  |  |  |  |  |  |
| Intubation before arrival or in emergency room | 0.46*** | 0.19*** | 0.19*** | 0.45*** | 0.15*** | 0.17*** | 0.15*** | 0.13*** | 0.07* | 0.08** | 0.01 | 0.03 | 0.07* | 0.1*** |
| SaO2 < 90% | 0.25*** | 0.16*** | 0.19*** | 0.12*** | 0.12*** | 0.12*** | 0.14*** | 0.17*** | 0.04 | 0.07* | -0.02 | 0.01 | 0.03 | 0.13*** |
| Systolic (BP < 90 mmHg) | 0.16*** | 0.14*** | 0.13*** | 0.1*** | 0.18*** | 0.19*** | 0.1*** | 0.06* | 0.05 | 0.11*** | 0.04 | 0.04 | 0.08** | 0.08** |
| 1st GCS < 14 | 0.37*** | 0.07* | 0.08** | 0.57*** | 0.06 | 0.05 | 0.07* | 0.07* | 0 | 0.05 | 0.01 | 0 | 0.04 | 0.01 |
| Combined physiological criteria (1st GCS < 14, intubation, SaO2 < 90%, BP < 90 mmHg) | 0.39*** | 0.13*** | 0.14*** | 0.47*** | 0.1*** | 0.09*** | 0.11*** | 0.1*** | 0.05 | 0.06* | 0.01 | 0.01 | 0.05 | 0.07* |
| ***Overall combined criteria*** |  |  |  |  |  |  |  |  |  |  |  |  |  |  |
| Overall Emergency room criterion | 0.33*** | 0.19*** | 0.18*** | 0.25*** | 0.09*** | 0.08** | 0.19*** | 0.17*** | 0.11*** | 0.07* | 0.07* | 0.04 | 0.11*** | 0.12*** |

*^1^Braken P, Amsler F, Gross T. Simple modification of trauma mechanism alarm criteria published for the TraumaNetwork DGU® may significantly improve overtriage – a cross sectional study. Scand J Trauma Resusc Emerg Med. BioMed Central; 2018;26:32.*

ISS, injury severity score; BP, blood pressure; SaO2, blood oxygen saturation; GCS, Glasgow Coma Scale, EMS injury emergency medical service’s grading of injury severity: at least one grade severe or two moderate injuries.

(The power of the statistical interrelation and significance (*t* and *p*, respectively) is illustrated by the intensity of the particular color used.)
